# Osteoarticular Tuberculosis

**DOI:** 10.4269/ajtmh.21-1258

**Published:** 2022-05-09

**Authors:** Juan Cataño, Julian Sanchez-Bautista

**Affiliations:** ^1^ Section of Infectious Diseases, Department of Internal Medicine, University of Antioquia School of Medicine, Medellín, Colombia;; ^2^ Department of Internal Medicine, University of Antioquia School of Medicine, Medellín, Colombia

**Figure 1.**
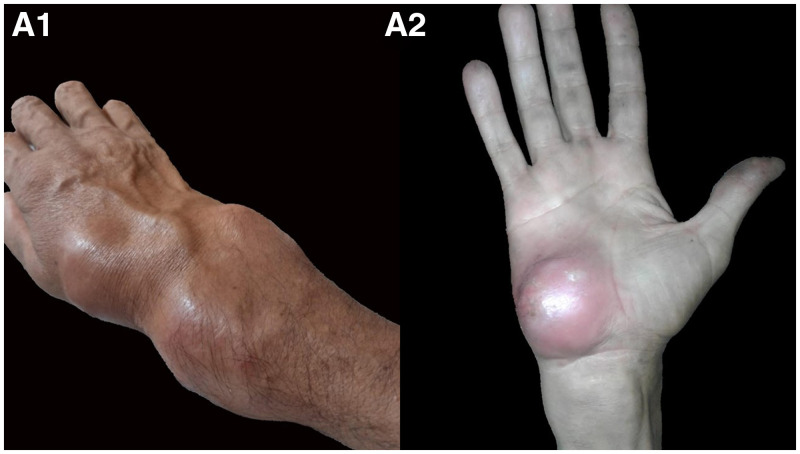
Right upper limb deformities on the dorsum of the hand, the thenar eminence, the posterior radial edge of the distal forearm, and the base of the first finger. This figure appears in color at www.ajtmh.org.

**Figure 2.**
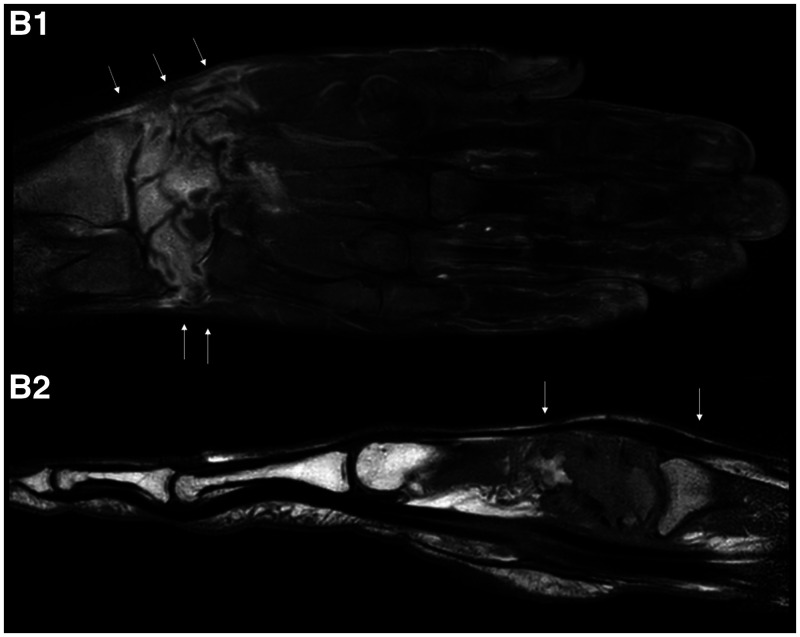
Contrast-enhanced magnetic resonance images of the hand showing synovitis, bone erosion, and pseudo-tumors extending up to the distal third of the forearm.

A 42-year-old man with no relevant past medical history, nor use of any immunosuppressing agents, presented with a 2-year history of right upper limb deformities on the dorsum of the hand, the thenar eminence, the posterior radial edge of the distal forearm, and the base of the first finger with wrist movement limitation (Figure 1). He denied any prior history of tuberculosis (TB) or any known TB contact. The patient’s main symptoms were related to his hand, but he also mentioned a chronic dry cough, with no fever, weight loss, or night sweats. Contrast-enhanced MRI of the hand showed synovitis, bone erosion, and pseudo-tumors extending up to the distal third of the forearm (Figure 2). The patient was taken directly into surgery, where abscess drainage was performed, obtaining 20 mL of pus, and bone and synovial biopsies were performed. Histologic analysis showed liquefaction necrosis, granulomatous inflammation, abundant acid-fast bacilli on Ziehl-Neelsen staining, and a positive TB polymerase chain reaction (PCR) test in all samples. Accordingly, a test for HIV was performed and the results were negative. Chest X-ray showed a reticular and micronodular pattern consistent with miliary TB, which was later confirmed by sputum TB-PCR. Firstline treatment was started with rifampicin, isoniazid, pyrazinamide, and ethambutol, according to local guidelines, which eventually improved most of the patient’s symptoms. The patient was discharged 1 week later, with minimal wrist movement limitation.

TB is a major public health problem in developing countries, where extrapulmonary TB is known to have many and varied presentations. Extraspinal tuberculous osteomyelitis is rare and comprises only 2% to 3% of all cases of osteoarticular TB, with the hip and knee joints being the most commonly involved. Wrist TB is rare, accounting for less than 1% of all cases of bone TB.^1^ Early diagnosis of this disease is difficult, and therefore the misdiagnosis rate is quite high. It takes an average of 16 to 19 months from the onset of carpal symptoms to a clear diagnosis.^2^ Subtle clinical and unremarkable radiographic features are the main reasons for a delayed diagnosis of TB. Early diagnosis and treatment may help to restore the function of the affected limb and thereby improve quality of life,^3^ but it is important to emphasize the relevance of aggressive debridement as a fundamental part of the treatment to avoid recurrence of infection with a very high organism burden, where is more likely to develop resistance, with catastrophic epidemiological consequences. Our case emphasizes the importance of making extrapulmonary TB an important differential diagnosis even in cases with clinical features that are completely inconsistent with tubercular infections. This is particularly applicable to countries that are TB endemic.
